# Physical therapy interventions for cervicogenic dizziness in a military-aged population: protocol for a systematic review

**DOI:** 10.1186/s13643-020-01335-4

**Published:** 2020-03-23

**Authors:** Carrie W. Hoppes, Anthony J. Romanello, Kathryn E. Gaudette, William K. Herron, Anne E. McCarthy, Catherine J. McHale, Joan Bares, Rose Turner, Susan L. Whitney

**Affiliations:** 1 Army-Baylor University Doctoral Program in Physical Therapy, Joint Base San Antonio-Fort Sam Houston, TX 78234 USA; 2 Stimson Library, U.S. Army Medical Center of Excellence, Joint Base San Antonio-Fort Sam Houston, TX USA; 3 Falk Library, University of Pittsburgh, Pittsburgh, PA USA; 4 Department of Physical Therapy, University of Pittsburgh, Pittsburgh, PA USA

**Keywords:** Systematic review, Cervicogenic dizziness, Physical therapy, Military

## Abstract

**Background:**

Traumatic cervicogenic dizziness is dizziness that is temporally associated with neck pain and injury after other causes of dizziness have been excluded. It can lead to activity limitations and participation restrictions that may include lost duty or work days. The objective of this systematic review is to determine which interventions are most effective in decreasing dizziness or vertigo and neck pain in military-aged adults with traumatic cervicogenic dizziness.

**Methods:**

The literature will be systematically searched using the following online databases: MEDLINE, EMBASE, The Cochrane Library (Cochrane Database of Systematic Reviews, CENTRAL, Cochrane Methodology Register), CINAHL, SCOPUS, Web of Science, and J-STAGE. The review will include randomized controlled trials (RCTs), including cluster RCTs and controlled (non-randomized) clinical trials or cluster trials, and observational studies (including prospective and retrospective comparative cohort and case–control or nested case–control studies) and determine the effectiveness of physical therapy interventions for the treatment of traumatic cervicogenic dizziness in military-aged adults. Assessment of methodological quality will be performed by two independent, blinded reviewers using the PEDro scale. The level of evidence will be determined using the GRADE scale. The primary outcome measures will be change in dizziness and neck pain and disability from baseline to the last available follow-up, measured using the Dizziness Handicap Inventory and Neck Disability Index. Other relevant outcome measures will include self-reported change in symptoms, time to return to duty or work, and quality of life.

**Discussion:**

This systematic review will identify, evaluate, and integrate the evidence on the effectiveness of physical therapy interventions for cervicogenic dizziness in a military-aged population. We anticipate our findings may inform individual treatment and future research. Clinical recommendations generated from this systematic review may inform military physical therapy treatment of individuals with cervicogenic dizziness.

**Systematic review registration:**

In accordance with the guidelines, our systematic review protocol was registered with the International Prospective Register of Systematic Reviews (PROSPERO) on 21 January 2020 (registration number CRD42020150853). In the event of protocol amendments, the date of each amendment will be accompanied by a description of the change and the rationale.

## Background

Dizziness is a common, non-specific symptom and is among one of the most frequent medical complaints [1]. Defined as disorientation that produces a disturbed postural awareness, dizziness has been attributed to neurologic, vestibular, psychosomatic, and cervical spine dysfunction [2, 3]. Of these etiologies, this systematic review is concerned specifically with dizziness related to cervical spine dysfunction. First defined by Ryan and Cope in 1955, dizziness originating from the cervical spine, or cervicogenic dizziness (CGD), is a symptom that is produced by changes in the position of the neck [4]. Over 30% of CGD cases are caused by flexion–extension injuries or whiplash [5]. According to Reid et al., whiplash injuries are experienced by 0.1% of the general population, with resultant dizziness being reported at rates ranging from 20 to 90% [6]. Currently, little is known about the incidence of CGD in the military. A retrospective study of active-duty military patients treated for dizziness after mild traumatic brain injury found a subset of patients that benefited from specific cervical spine proprioceptive training [7].

Cervicogenic dizziness can result in a variety of coexisting symptoms such as unsteadiness, lightheadedness, perceptions of spinning, nausea, and general disorientation [8]. Thompson-Harvey and Hain reported that patients with cervical vertigo have neck pain (94%), true vertigo (27%), and headache (50%) [9]. These disabling symptoms may have significant psychological repercussions which can lead to anxiety, depression, and difficulty completing activities of daily living and occupational duties [8]. Patients with both dizziness and neck pain have a lower perceived mental and physical quality of life when compared to patients with dizziness only [10]. Spitzer et al. reported that 20% of those who experience whiplash injuries are unable to return to work for more than 20 weeks [11].

The underlying pathogenesis of traumatic CGD has been attributed to damaged joint receptors within the upper cervical spine (C1–C3) causing abnormal afferent input to the vestibular nuclei [4]. The proprioceptive system contained in these joint capsules has an abundance of mechanoreceptors which supply afferent information concerning the orientation of the head relative to the rest of the body [3, 4]. This system can become damaged due to direct trauma, muscular fatigue, or degeneration and may result in CGD [3]. Cervical spine pathology may disrupt the strong connections between the cervical dorsal roots and the vestibular nuclei that contribute to the perception of balance and postural adjustments, leading to complaints of dizziness or disequilibrium [12]. Ataxia and a strong sensation of ipsilateral falling or tilting can be induced by injecting local anesthetics into the neck, presumably interrupting afferent input from neck muscle and joint receptors [13].

According to a recent systematic review [8], manual therapy is the most common form of treatment for CGD. Several systematic reviews have explored the effects of manual therapy on patients with CGD with several randomized controlled trials having evaluated varying manual techniques, such as sustained natural apophyseal glides (SNAGs) [6, 8, 14]. According to Reid et al. [6], while manual therapy may improve the signs and symptoms of CGD, there is still a need for higher quality evidence. Manual therapy used in conjunction with vestibular rehabilitation as well as without was reviewed by Lystad et al. in a systematic review [14]. This form of rehabilitation consists of movement-based exercises focused on maximizing central nervous system compensation for peripheral vestibular disorders [14]. However, there were no studies that combined vestibular rehabilitation exercises in addition to manual therapy; therefore, this systematic review was not able to establish vestibular rehabilitation in conjunction with manual therapy as a supported treatment for CGD.

Yaseen et al. [8] produced the most recent systematic review, with only four studies meeting their inclusion criteria. These studies [15–18], all of which focused on manual therapy, reported positive outcomes on various measures, such as the Dizziness Handicap Inventory (DHI), visual analog scale (VAS) for dizziness, and frequency of dizziness. Maitland passive mobilizations and Mulligan SNAGs did not produce a significant difference in intensity of dizziness when compared to placebo after 12 months; however, there was a significant difference in frequency of dizziness and DHI for both passive mobilizations and SNAGs [18]. The systematic reviews by Reid [6] and Yaseen [8] both suggest that there is a need for higher quality evidence to inform the use of manual therapy as a treatment technique for CGD, and Lystad et al. [14] recommends further researching manual therapy in conjunction with vestibular rehabilitation as a possible treatment for CGD.

The 2018 Health of the Force Report [19] found that 95% of active-duty US Army soldiers are 18–45 years old and 85% are male. Within three systematic reviews [6, 8, 14], only four of the included RCTs [20–23] applied to the military-aged population. Of these four RCTs, three compared spinal manipulation to a control group [20–22], while the other compared a multimodal intervention consisting of soft tissue therapy, mobilizations, home training programs, and body awareness to a delayed treatment group who later received the same interventions and healthy controls [23]. The multimodal intervention was individualized for each subject [23]; without a standardized treatment protocol, clinicians cannot replicate this treatment approach. All four RCTs noted improvements in outcome measures (DHI, VAS, and reduced frequency and duration of dizziness). However, none of the four RCTs was conducted in a military population. Another gap in the current literature is the relatively small sample sizes and skewed gender distributions used in these RCTs. Of the RCTs included in the most recent systematic reviews, two included a predominately female population [16, 18]. This contrasts from the predominantly male population of the US military.

The present study differs from previous systematic reviews in several ways. Previously reported interventions were provided within the healthcare setting, but the current study will focus on reviewing interventions that are feasible in austere or far-forward environments and involve military or analogous populations. Manual therapy is the primary intervention utilized in past systematic reviews for the management of CGD; however, this study aims to include a variety of interventional approaches such as education, exercise, manual therapy, vestibular rehabilitation, and modalities for the management of CGD. Lastly, in addition to the DHI and VAS, returning to duty or work and quality of life will be included as outcome measures. The objective of this systematic review is to determine which interventions are most effective in decreasing dizziness or vertigo and neck pain in military-aged adults with traumatic CGD. Our secondary aim will be to generate clinical recommendations for military physical therapists treating individuals with CGD.

## Methods/design

The present protocol has been registered within the PROSPERO database (registration number CRD42020150853) and is being reported in accordance with the reporting guidance provided in the Preferred Reporting Items for Systematic Reviews and Meta-Analyses Protocols (PRISMA-P) statement [24] (see checklist in Additional file 1).

### Eligibility criteria

Studies will be selected according to the criteria outlined below.

#### Study designs

We will include RCTs, including cluster RCTs and controlled (non-randomized) clinical trials (CCTs) or cluster trials, to assess the beneficial effects of the interventions. We will supplement these with observational studies (including prospective and retrospective comparative cohort and case–control or nested case–control studies). We will exclude cross-sectional studies, case series, and case reports. Study protocols and abstract-only records will also be excluded.

#### Participants

We will include studies treating military-aged adult humans (18-45 years old) with traumatic CGD. If most, but not all, of the study population is under 45 years old, the study will be included. We will exclude studies treating degenerative cervical spine disorders, Barré–Liéou syndrome, Bow Hunter’s syndrome, and Beauty Parlor syndrome [25]. Studies of whiplash without associated dizziness will be excluded. Studies treating other types of dizziness (those related to the ear, nose, and throat; central nervous system; and cardiovascular system) will also be excluded. For example, we will exclude studies on benign paroxysmal positional vertigo, perilymphatic fistula, labyrinthine concussion, unilateral and bilateral peripheral vestibular hypofunction, vestibular migraine, and traumatic brain injury.

#### Interventions

Physical therapy interventions must be conducive to an austere or far-forward (close to the battlefield) military environment. This may be a combat zone where physical therapists treat patients in harsh and hostile environments with little to no equipment or supplies [26]. This will limit interventions to techniques and equipment that is ruggedized (designed to be hard-wearing and/or shock-resistant) and portable. Examples include exercise, manual therapy (mobilization and manipulation), and dry needling. We will classify interventions described in studies according to the following broad categories: education, exercise, manual therapy, vestibular rehabilitation, and modalities. Interventions may be used in isolation or in combination.

#### Comparators

Given the broad prospective for interventions of interest, several comparisons will be relevant to include. These may include placebo, usual care, higher versus lower intervention dosage, or different types of interventions applied with similar dosage.

#### Outcomes

The primary outcome measures will be change in dizziness and neck pain and disability from baseline to the last available follow-up, measured using the DHI [27] and Neck Disability Index [28]. Secondary outcome measures will include other scales of self-reported change in symptoms (to include severity, frequency, and duration) such as verbal or visual analog scales for dizziness or neck pain. Tertiary outcome measures will include time to return to duty or work and quality of life.

#### Timing

Studies will be selected for inclusion based on the length of follow-up of outcomes. All study designs should have a follow-up of at least 1 week.

#### Setting

There will be no restrictions by type of setting.

#### Language

We will include articles reported in the English and Japanese languages. The primary language of the authors is English, and one author (W.K.H.) can additionally read and speak Japanese.

### Information sources

Literature search strategies will be developed using subject headings and text words related to interventions for CGD. The draft search strategy for MEDLINE is presented in Additional file 2. The search terms will be adapted for use with other bibliographic databases. For J-STAGE, the search strategy is 頸椎めまい (頸椎 cervical vertebrae region; めまい dizziness). We will search the following electronic bibliographic databases: MEDLINE (PubMed and OVID interface), EMBASE (EMBASE.com interface), The Cochrane Library (Cochrane Database of Systematic Reviews, Cochrane Central Register of Controlled Trials [CENTRAL], Cochrane Methodology Register), Cumulative Index for Nursing and Allied Health Literature (CINAHL), SCOPUS, Web of Science (Science and Social Science Citation Index), and J-STAGE. PROSPERO will be searched for ongoing or recently completed systematic reviews. We will contact study authors as needed to request missing studies.

The literature search will be restricted to published studies in the English and Japanese languages and human subjects. As Ryan and Cope [4] first described dizziness originating from the cervical spine in 1955, studies published between January 1955 and the date the searches are run will be sought. The searches will be re-run immediately before the final analyses and further studies retrieved for inclusion. To ensure literature saturation, we will scan the reference lists of included studies or relevant reviews identified through the search.

### Study records

#### Data management

We will implement the search strategies and import all references identified into EndNote X9 (Clarivate; Philadelphia, PA). The search results from the different bibliographic databases will be combined in a single EndNote library, and we will remove duplicate articles by title and/or abstract. An online technology platform (Covidence; Melbourne, Australia) will be used to manage records and data throughout the review.

#### Study selection/selection process

Titles and/or abstracts of studies retrieved using the search strategy and those from additional sources will be independently screened by two review authors to identify studies that potentially meet the inclusion criteria outlined above. The full text of these potentially eligible studies will be retrieved and independently assessed for eligibility by two review authors. Any disagreement will be resolved through discussion with a third review author. Agreement between the two reviewers will be assessed using the Kappa statistic.

#### Data collection process

A standardized form will be used to extract data from the included studies for assessment of study quality and evidence synthesis. The extracted information will include study setting, study population and participant demographics and baseline characteristics, details of the intervention and comparison conditions, study methodology, study completion rates, outcomes and times of measurement, indicators of acceptability to users, suggested mechanisms of intervention action, and information for assessment of the risk of bias. Two review authors will extract data independently; discrepancies will be identified and resolved through discussion (with a third review author where necessary). Missing data will be requested from study authors.

### Data items

Participants must be military-aged adult humans (18–45 years old) with traumatic CGD, defined as dizziness that is temporally associated with neck pain and injury after other causes of dizziness (such as central or peripheral vestibular pathologies) have been excluded [29]. This review will include all studies examining at least one of the following interventions: (1) education (i.e., patient education, postural or ergonomic information, brochures), (2) exercise (i.e., cervical or scapular retraction, stabilization or strengthening, proprioceptive or kinesthetic retraining), (3) manual therapy (i.e., mobilization, manipulation, Mulligan or Maitland techniques, massage, myofascial release, suboccipital release), (4) vestibular rehabilitation (i.e., gaze stabilization, habituation), and (5) modalities (i.e., dry needling, acupuncture). Comparison interventions may include placebo, usual care, higher versus lower intervention dosage, or different types of interventions applied with similar dosage.

### Outcomes and prioritization

Primary outcome measures will include the DHI [27] and Neck Disability Index [28]. Secondary outcome measures will include other scales of self-reported change in symptoms (to include severity, frequency, and duration) such as verbal or visual analog scales for dizziness or neck pain. Tertiary outcome measures will include time to return to duty or work and quality of life.

### Risk of bias in individual studies

Two review authors will independently assess the risk of bias in included studies using the PEDro scale [30]. The 11-item PEDro Scale assesses the methodological quality of RCTs [30]. Disagreements between the review authors over the risk of bias will be resolved through discussion (with a third review author where necessary). Agreement between the two reviewers will be assessed using the Kappa statistic.

### Data synthesis

Data will be collected from the articles accepted in the study by two members of the research team. Both members are required to agree upon the selected data for them to be included. Data will be chosen based on their usefulness in determining the most effective intervention(s) in decreasing dizziness or vertigo and neck pain in military-aged adults with traumatic CGD. We will group interventions into education, exercise, manual therapy, vestibular rehabilitation, and modalities. A narrative synthesis of the findings from the included studies, structured around the target population (study sample) characteristics, type of intervention(s), treatment parameters, and type of outcome measures of the selected studies will be provided. Where such data are not presented in the original research article, the corresponding author will be contacted to retrieve unavailable and unclear data. Where available, *p* values of within-group changes from pre- to post-test for the outcome measures will be reported in summary tables. Cohen's *D* effect sizes from individual studies will be calculated for within-group changes from pre- to post-test for the outcome measures when such data are available. Effect size will be classified as described by Cook for interpretation of the results [31]. This quantitative analysis will provide the basis for the formal narrative synthesis. There is not a consensus on diagnostic criteria for CGD, and clinical heterogeneity can be expected because of variations in a population’s characteristics and applications of interventions between studies (i.e., frequency, intensity). Therefore, performance of a meta-analysis is not planned.

Summaries of intervention effects for each study will be provided by calculating standardized mean differences (for continuous outcomes) and effect sizes when possible.

The Grading of Recommendations, Assessment, Development and Evaluations (GRADE) scale [32] will be used to assess the strength of the body of evidence. There is not a consensus on diagnostic criteria for CGD, and clinical heterogeneity can be expected because of variations in a population’s characteristics and applications of interventions between studies (i.e., frequency, intensity). Therefore, performance of a meta-analysis is not planned.

### Meta-biases

There are no planned assessments of meta-biases due to publication bias across studies. Meta-biases due to selective reporting within studies will be assessed by (1) comparing outcomes reported in the protocol to those in the published report and/or (2) comparing outcomes reported in the methods and results sections of the published report.

### Additional analyses

This is a qualitative synthesis, and while subgroup analyses may be undertaken, it is not possible to specify the groups in advance. If a characteristic for treating traumatic CGD in a military-aged population was overlooked in this protocol but is clearly of major importance and justified by external evidence, we will explore it and report the subgroup analyses as post hoc [33].

## Discussion

This systematic review will be performed to critically examine the literature on the treatment of CGD. Specifically, we aim to determine which interventions are most effective in decreasing dizziness or vertigo and neck pain in military-aged adults with traumatic CGD. Understanding which interventions or combinations of interventions are most effective in decreasing dizziness or vertigo and neck pain may speed return to duty or work. In the future, findings from this review may support the generation of clinical recommendations for military physical therapists treating individuals with CGD.

This present review will focus only on the treatment of traumatic CGD in a military-aged population. Although this scope is narrowly focused, excluding CGD in older adults and CGD due to degenerative cervical spine, sympathetic, and vascular disorders, it is unique in including many types of interventions. There is a possibility that there will be too few studies available to draw valid conclusions from if the focus is on military-aged adults and treatments and techniques that are conducive to an austere or far-forward military environment. This review will be limited to the English and Japanese languages, which may introduce the risk of publication bias. At the individual study level, clinical heterogeneity can be expected. There is not a consensus on diagnostic criteria for CGD. Differences in acuity and severity of symptoms may exist, and applications of interventions between studies will vary. The possibility that interventions may have been developed and conducted by the same research groups and this potential source of bias will also be considered. Despite these limitations, this systematic review is important for identifying evidence-based interventions for decreasing dizziness or vertigo and neck pain in military-aged adults with traumatic CGD.

The results of this systematic review could help inform future research in the field of vestibular rehabilitation. Clinical subtypes may exist within CGD, and identifying which interventions are most effective in decreasing dizziness or vertigo and neck pain in adults with traumatic CGD could form the basis for RCTs exploring the timing and dosing of treatment(s). Identifying patient-related factors for responsiveness to these interventions would aid in matching the right patient to the right treatment. Clinical recommendations for physical therapists treating individuals with traumatic CGD arising from this review should be validated by future research.

Important amendments to this protocol will be updated within the PROSPERO database and documented in the full review.

## Supplementary information

**Additional file 1:.** PRISMA-P 2015 Checklist

**Additional file 2:.** The draft search strategy for MEDLINE to determine which interventions are most effective in decreasing dizziness or vertigo and neck pain in military-aged adults with cervicogenic dizziness

## Data Availability

The datasets used and/or analyzed during the current study are available from the corresponding author on reasonable request.

## Abbreviations

CCTs: Controlled clinical trials
CGD: Cervicogenic dizziness
CINAHL: Cumulative Index for Nursing and Allied Health Literature
DHI: Dizziness Handicap Inventory
GRADE: Grading of Recommendations, Assessment, Development and Evaluations
PRISMA-P: Preferred Reporting Items for Systematic Reviews and Meta-Analyses Protocols
PROSPERO: International Prospective Register of Systematic Reviews
RCTs: Randomized controlled trials
SNAGs: Sustained natural apophyseal glides
VAS: Visual analog scale

## References

[1] Yardley L, Owen N, Nazareth I, Luxon L (1998). Prevalence and presentation of dizziness in a general practice community sample of working age people. Br J Gen Pract.

[2] Strupp M, Brandt T (2008). Diagnosis and treatment of vertigo and dizziness. Dtsch Arztebl Int.

[3] Li Y, Peng B (2015). Pathogenesis, diagnosis, and treatment of cervical vertigo. Pain Physician.

[4] Ryan GM, Cope S (1955). Cervical vertigo. Lancet.

[5] Hulse M (1982). Differential diagnosis of vertigo in functional cervical vertebrae joint syndromes and vertebrobasilar insufficiency. HNO.

[6] Reid SA, Rivett DA (2005). Manual therapy treatment of cervicogenic dizziness: a systematic review. Man Ther.

[7] Hammerle M, Swan AA, Nelson JT, Treleaven JM (2019). Retrospective review: effectiveness of cervical proprioception retraining for dizziness after mild traumatic brain injury in a military population with abnormal cervical proprioception. J Manipulative Physiol Ther.

[8] Yaseen K, Hendrick P, Ismail A, Felemban M, Alshehri MA (2018). The effectiveness of manual therapy in treating cervicogenic dizziness: a systematic review. J Phys Ther Sci.

[9] Thompson-Harvey A, Hain TC (2019). Symptoms in cervical vertigo. Larynscope Investig Otol.

[10] Kalland Knapstad M, Goplen F, Skouen JS, Ask T, Nordahl SHG. Symptom severity and quality of life in patients with concurrent neck pain and dizziness. Disabil Rehabil. 2019:1–4. 10.1080/09638288.2019.1571640 30739502

[11] Spitzer WO, Skovron ML, Salmi LR, Cassidy JD, Duranceau J, Suissa S (1995). Scientific monograph of the Quebec Task Force on Whiplash-Associated Disorders: redefining "whiplash" and its management. Spine (Phila Pa 1976).

[12] Brown JJ. Cervical contributions to balance: cervical vertigo. New York, N.Y.: Oxford University Press; 1992.

[13] de Jong PT, de Jong JM, Cohen B, Jongkees LB. Ataxia and nystagmus induced by injection of local anesthetics in the neck. Ann Neurol. 1977;1(3):240–6. 10.1002/ana.410010307 407834

[14] Lystad RP, Bell G, Bonnevie-Svendsen M, Carter CV. Manual therapy with and without vestibular rehabilitation for cervicogenic dizziness: a systematic review. Chiropr Man Therap. 2011;19(1):21. 10.1186/2045-709X-19-21 PMC3182131 21923933

[15] Malmström E, Karlberg M, Melander A, Magnusson M, Moritz U. Cervicogenic dizziness - musculoskeletal findings before and after treatment and long-term outcome. Disabil Rehabil. 2007;29(15):1193–205. 10.1080/09638280600948383 17653993

[16] Reid SA, Rivett DA, Katekar MG, Callister R (2008). Sustained natural apophyseal glides (SNAGs) are an effective treatment for cervicogenic dizziness. Manual Therapy.

[17] Reid SA, Rivett DA, Katekar MG, Callister R. Comparison of Mulligan sustained natural apophyseal glides and Maitland mobilizations for treatment of cervicogenic dizziness: a randomized controlled trial. Physical Therapy. 2014;94(4):466–76. 10.2522/ptj.20120483 24336477

[18] Reid SA, Callister R, Snodgrass SJ, Katekar MG, Rivett DA (2015). Manual therapy for cervicogenic dizziness: long-term outcomes of a randomised trial. Manual Therapy.

[19] US Army Public Health Center. 2018 Health of the Force Report [Available from: https://phc.amedd.army.mil/Periodical%20Library/2018HealthoftheForceReport.pdf.

[20] Du HG, Wei H, Huang MZ, Jiang Z, Ye SL, Song HQ (2010). Randomized controlled trial on manipulation for the treatment of cervical vertigo of high flow velocity type. Zhongguo Gu Shang.

[21] Fang J (2010). Observation of curative effect on fixed-point spin reduction of spinal manipulation therapy for cervical vertigo. Zhongguo Gu Shang.

[22] Kang F, Wang QC, Ye YG (2008). A randomized controlled trial of rotatory reduction manipulation and acupoint massage in the treatment of younger cervical vertigo. Zhongguo Gu Shang.

[23] Karlberg M, Magnusson M, Malmstrom EM, Melander A, Moritz U (1996). Postural and symptomatic improvement after physiotherapy in patients with dizziness of suspected cervical origin. Arch Phys Med Rehabil.

[24] Shamseer L, Moher D, Clarke M, Ghersi D, Liberati A, Petticrew M, et al. Preferred reporting items for systematic review and meta-analysis protocols (PRISMA-P) 2015: elaboration and explanation. BMJ. 2015;349. 10.1136/bmj.g7647 25555855

[25] Devaraja K (2018). Approach to cervicogenic dizziness: a comprehensive review of its aetiopathology and management. Eur Arch Otorhinolaryngol.

[26] Moore JH, Goffar SL, Teyhen DS, Pendergrass TL, Childs JD, Ficke JR (2013). The role of US military physical therapists during recent combat campaigns. Physical Therapy.

[27] Jacobson GP, Newman CW (1990). The development of the Dizziness Handicap Inventory. Arch Otolaryngol Head Neck Surg.

[28] Vernon H, Mior S (1991). The Neck Disability Index: a study of reliability and validity. J Manipulative Physiol Ther.

[29] Wrisley DM, Sparto PJ, Whitney SL, Furman JM (2000). Cervicogenic dizziness: a review of diagnosis and treatment. J Orthop Sports Phys Ther.

[30] Physiotherapy Evidence Database. PEDro Scale [Available from: https://www.pedro.org.au/english/downloads/pedro-scale/.

[31] Cook C (2008). Clinimetrics corner: use of effect sizes in describing data. J Man Manipulative Therapy.

[32] Guyatt GH, Oxman AD, Vist GE, Kunz R, Falck-Ytter Y, Alonso-Coello P (2008). GRADE: an emerging consensus on rating quality of evidence and strength of recommendations. BMJ.

[33] Cochrane Handbook for Systematic Reviews of Interventions: The Cochrane Collaboration; 2011 [Version 5.1.0: Available from: https://handbook-5-1.cochrane.org/front_page.htm.

