# Supplementary material for: Physical therapy interventions for cervicogenic dizziness in a military-aged population: protocol for a systematic review
Source: Syst Rev. 2020 Mar 23;9:62. doi: 10.1186/s13643-020-01335-4 (PMC7087372; doi:10.1186/s13643-020-01335-4)
Supplement: Supplementary file 2 — Additional file 2:. The draft search strategy for MEDLINE to determine which interventions are most effective in decreasing dizziness or vertigo and neck pain in military-aged adults with cervicogenic dizziness [file 13643_2020_1335_MOESM2_ESM.docx]

**ADDITIONAL FILE 2**

The draft search strategy for MEDLINE to determine which interventions are most effective in decreasing dizziness or vertigo and neck pain in military-aged adults with cervicogenic dizziness:

(((((((((((((((((((physical therapy modalities[MeSH Terms]) OR "Exercise Therapy"[Mesh]) OR proprioception[MeSH Terms]) OR posture[MeSH Terms]) OR "Postural Balance"[Mesh]) OR traction[MeSH Terms]) OR patient education as topic[MeSH Terms]) OR acupuncture therapy[MeSH Terms]) OR acupuncture[MeSH Terms]) OR "Needles"[Mesh]) OR central muscle relaxants[MeSH Terms]) OR opioid analgesics[MeSH Terms]) OR agents, nonsteroidal antiinflammatory[MeSH Terms]) OR herbal medicine[MeSH Terms]) OR clozapine[MeSH Terms]) OR benzodiazepines[MeSH Terms])) OR (((((((((((((((((((((((((((((((physical therap*[Title/Abstract]) OR physiotherap*[Title/Abstract]) OR rehabilitation[Title/Abstract]) OR manipulation[Title/Abstract]) OR manual therap*[Title/Abstract]) OR exercise therap*[Title/Abstract]) OR massage[Title/Abstract]) OR mulligan[Title/Abstract]) OR maitland[Title/Abstract]) OR myofascial[Title/Abstract]) OR transcutaneous electric nerve stimulation[Title/Abstract]) OR scapular retraction[Title/Abstract]) OR stabilization[Title/Abstract]) OR suboccipital release[Title/Abstract]) OR propriocept*[Title/Abstract]) OR postur* balance[Title/Abstract]) OR postur* instability[Title/Abstract]) OR vestibular rehabilitation[Title/Abstract]) OR gaze stabili*[Title/Abstract]) OR traction[Title/Abstract]) OR patient education[Title/Abstract]) OR acupuncture[Title/Abstract]) OR dry needling[Title/Abstract]) OR muscle relaxant*[Title/Abstract]) OR opioid*[Title/Abstract]) OR non-steroid*[Title/Abstract]) OR herb*[Title/Abstract]) OR herb*[Title/Abstract]) OR clozapine[Title/Abstract]) OR dibenzodiazepine[Title/Abstract]) OR benzodiazepines[Title/Abstract]))) AND (((((((neck pain[Title/Abstract]) OR neck injur*[Title/Abstract]) OR whiplash*[Title/Abstract]) OR cervic*[Title/Abstract]) OR flexion extension injur*[Title/Abstract])) AND ((((dizziness[Title/Abstract]) OR dizzyness[Title/Abstract]) OR vertigo[Title/Abstract]) OR lightheadedness[Title/Abstract]))
